# Exosomal MiRNAs in Pediatric Cancers

**DOI:** 10.3390/ijms20184600

**Published:** 2019-09-17

**Authors:** Angela Galardi, Marta Colletti, Virginia Di Paolo, Patrizia Vitullo, Loretta Antonetti, Ida Russo, Angela Di Giannatale

**Affiliations:** Department of Pediatric Hematology/Oncology, IRCCS, Ospedale Pediatrico Bambino Gesù, 00146 Rome, Italy; angela.galardi@opbg.net (A.G.); virginia.dipaolo@opbg.net (V.D.P.); patrizia.vitullo@opbg.net (P.V.); loretta.antonetti@opbg.net (L.A.); ida.russo@opbg.net (I.R.); angela.digiannatale@opbg.net (A.D.G.)

**Keywords:** exosomes, miRNAs, biomarkers, pediatric cancer

## Abstract

MicroRNAs (miRNAs) have generated great attention in oncology as they play a fundamental role in the regulation of gene expression and their aberrant expression is present in almost all types of tumors including pediatric ones. The discovery that miRNAs can be transported by exosomes, which are vesicles of 40–120 nm involved in cellular communication, that are produced by different cell types, and that are present in different biological fluids, has opened the possibility of using exosomal miRNAs as biomarkers. The possibility to diagnose and monitor the progression and response to drugs through molecules that can be easily isolated from biological fluids represents a particularly important aspect in the pediatric context where invasive techniques are often used. In recent years, the idea of liquid biopsy as well as studies on the possible role of exosomal miRNAs as biomarkers have developed greatly. In this review, we report an overview of all the evidences acquired in recent years on the identification of exosomal microRNAs with biomarker potential in pediatric cancers. We discuss the following herein: neuroblastoma, hepatoblastoma, sarcomas (osteosarcoma, Ewing’s sarcoma and rhabdoid tumors, and non-rhabdomyosarcoma soft tissue sarcoma), brain tumors, lymphomas, and leukemias.

## 1. Introduction

Tissue biopsy still represents the gold standard for tumor diagnosis. Nevertheless, minimally invasive approaches for detecting and monitoring tumors are needed in the pediatric setting. Liquid biopsies represent a less invasive source of biomarkers for patient monitoring and therapeutic decision. Indeed, the analysis of cell-free DNA, RNA, and soluble proteins is a potential alternative to the classic tumor biopsy [1]. However, a rigorous standardization and extensive validation are required before widespread use in routine clinical practice. Tumor cells constitutively release exosomes, nanovesicles of 40–120 nm, which reflect characteristics of the parental cell of origin and play an important role in cell–cell communication [2,3]. It is now well known that the number of exosomes released from neoplastic cells is increased compared to normal cells [4]; furthermore, qualitative and quantitative changes of their cargo are observed during tumor progression, reflecting tumor evolution [5,6,7]. Exosomes transport proteins, lipids, DNA, RNA, and miRNAs that represent potential biomarkers for clinical purposes. Among these, miRNAs have attracted great attention as they play a central role in gene-expression regulation and their expression is aberrant in cancer. The evidence that tumor cells communicate via the secretion of miRNAs in body fluids and that these molecules may be packed into exosomes and delivered to target cells has opened interesting scenarios for the use of these vesicles as new biomarkers. The miRNAs contained in the exosomes are particularly stable in biological fluids since they are protected by a double lipid layer. Moreover, exosomes contain specific repertoires of miRNAs, which are selectively sorted in these vesicles [8]. For this reason, exosomal miRNAs display different expression patterns between cancer patients and healthy individuals. Furthermore, miRNA expression profiles are related to tumor aggressiveness and may vary during tumor progression. The possibility to isolate exosomes from different biological fluids, such as blood and urine, as well as to monitor miRNAs in a relatively simple way allows for monitoring of tumor evolution and for evaluating the response to therapy over time. The purpose of this review is to summarize the recent supportive evidences on the potential role of exosomal miRNAs as biomarkers in pediatric cancers.

## 2. MiRNAs: Biogenesis and Functions

MiRNAs are single-stranded noncoding RNA molecules consisting of 18–20 nucleotides involved in posttranscriptional regulation of gene expression. MiRNAs bind target messenger RNA (mRNA) to prevent protein production by two distinct mechanisms: cleavage of mRNA with subsequent degradation or translation inhibition. MiRNAs are transcribed by RNA polymerases II and III, generating precursors of approximately 70 nucleotides in length (pre-miRNA) that undergo a series of cleavage events to form mature miRNA. The pre-miRNA is then translocated from the nucleus to the cytoplasm by exportin-5 and processed by the protein Dicer that cleaves pre-miRNA into short fragments. This process generates a mature miRNA, which incorporates into the effector complex RNA-induced silencing complex (RISC). The miRNA acts as a guide by base pairing with target mRNA and negatively regulates its expression [9]. MiRNAs are regulators of several biological processes, including embryonic and cellular development, cellular differentiation, proliferation, apoptosis, and metabolism [10,11,12]. The expression of miRNAs is finely tuned and highly specific and may be tissue-, cell-, or developmental-stage specific [13]. Moreover, a single miRNA can regulate many mRNAs and many miRNAs may regulate the expression of a single mRNA, thus generating a very complex regulatory network. Given their key role in the regulation of transcription and translation, deregulation of their expression and/or function has been associated with several diseases, including cancer. Prediction analyses of miRNA targets and gain- and loss-of-function experiments have elucidated the role of certain miRNAs in tumorigenesis. Indeed, the dysregulated miRNAs may act as either tumor suppressors or oncogenes in cancer by increasing proliferation, angiogenesis, invasion, and metastasis and by inhibiting apoptosis. 

## 3. Exosomal MiRNAs: Biogenesis, Sorting, and Function 

Exosomes originate from endosomal compartments through two phases: endocytosis of the plasma membrane that leads to the formation of early endosomes and inward budding from the outer membrane of endosomes. Cytosol elements are incorporated into the exosome lumen during their formation. The Endosomal Sorting Complexes Required for Transport (ESCRT) in cooperation with tumor-susceptibility gene 101 (TSG101) and ALG-2 interacting protein X (ALIX) is responsible for the internalization of ubiquitinated proteins into microvesicular bodies (MVB) [14]. ESCRT-0 recognizes and retains in the late endosomal membrane the ubiquinated proteins. ESCRT-I/ESCRT-II trigger involution of the limiting membrane into the MVB lumen, and ESCRT-III forms a spiral-shaped structure that constricts the budding neck, inducing ATPase VPS4 activation and membrane scission [15]. Sorting of proteins into MVB can also occur in a ubiquitin-independent way. 

The exosomal miRNA profile is a hallmark of tumor cell types, reflecting the status of parental cells [16,17]. The mechanism of miRNA incorporation in exosomes is highly specific: several studies have demonstrated that parent cells have a sorting mechanism that directs specific intracellular miRNAs to enter exosomes [18,19]. Although the underlying mechanisms remain unknown, it has been described that miRNAs can be sorted into exosomes by four possible paths: a) the neuronal sphingomyelinase 2 (nSMase)-dependent pathway: overexpression of nSMase2 increases the number of miRNAs embodied in exosomes while its inhibition reduces the number of exosomal miRNAs [20]; b) the miRNA motif and sumoylated heterogeneous nuclear ribonucleoproteins (hnRNPs)-dependent pathway: hnRNPA2B1 recognizes the GGAG motif in 3′ UTR of miRNA sequences, determining its packaging into exosomes [8]; c) the 3′ end of the miRNA sequence-dependent pathway: 3′ ends of uridylated endogenous miRNAs are mainly presented in exosomes derived from B cells or urine, whereas the 3′ ends of adenylated endogenous miRNAs are especially presented in B cells [21]; and d) the miRNA-induced silencing complex (miRISC)-related pathway: the knockout of argonaute protein 2 (AGO2) could decrease the types and abundance of preferentially exported miRNAs in exosomes isolated from HEK293T cells [18]. Given that the content of exosomal miRNAs change in different pathological conditions, including tumors, it would be important to investigate the alterations of the selective exosome loading in cancer. Indeed, the restoration of a normal selective loading of miRNAs in a cancer cell could lead to the correction of pathological messages that are sent to target cells through exosomes.

## 4. Role of Exosomal MiRNAs in Cancer

Intercellular communication is a key element of tumor progression and metastasis. Exosomes are involved in several aspects of tumorigenesis such as angiogenesis, chemoresistance, immunomodulation, and metastasis. In the tumor microenvironment, exosomes may indeed transport and transfer several bioactive molecules such as miRNAs, which can act as oncogenes as well as tumor suppressor genes. The induction of angiogenesis via exosomal miRNAs has been well documented in various cancers such as rhabdomyosarcoma [22]. Exosomal miR-17-92 cluster regulates gene expression during angiogenesis in leukemia [23]; in colon rectal cancer (CRC), exosomal miR-1246 promotes human umbilical vascular endothelial cell (HUVEC) proliferation, migration, and tube formation, thus activating the Smad 1/5/8 pathway [24]; and in breast cancer, miR-122 carried in exosomes mediates high levels of angiogenesis to sustain tumor growth and to facilitate metastasis [25].

Exosomal miRNAs are able to modulate drug susceptibility of cancer cells. It has been observed that exosomal miR-34a increases sensitivity of prostate cancer cells to docetaxel by regulating Bcl-2 [26]. Temozolomide (TMZ)-resistant glioblastoma (GBM) cells become sensitive to TMZ through the targeting of the X-ray repair cross-completing 4 (XRCC4) by exosomal miR-151a [27]. MiR-221/222 contained in exosomes released by tamoxifen-resistant breast cancer cells inhibits the expression of *p27* and estrogen receptor (ER)-α, conferring tamoxifen resistance in target cells [28]. These studies represent some examples of how exosomal miRNAs contribute to the development of drug resistance, leading to tumor escape and recurrence. 

Exosomal miRNAs are also critical mediators in the cross talk between cancer and immune cells. For example, it has been reported that adenocarcinoma cells produce exosomes containing miR-let-7a-5p, miR-10a-5p, miR-1246, and miR-125b-5p that promote macrophage (M) polarization toward a pro-inflammatory and antitumor M1 phenotype [29]. Conversely, miR-222-3p carried in exosomes derived from epithelial ovarian cancer cells produces exosomes which induce macrophage polarization and differentiation to the M2 phenotype promoting tumor growth and metastasis [30]. In addition to acting on macrophages, tumor-derived exosomal miRNAs drive disfunction of immune cells facilitating immunosuppression. Nasopharyngeal carcinoma cells secrete exosomes containing different miRNAs (miR-24-3p, miR-891a, miR-106a-5p, miR-20a-5p, and miR-1908) that inhibit T-cell proliferation, targeting mitogen-activated protein kinase (MAPK)-1 and signal transducer and activator of transcription (STAT) pathways [31]. The expression of regulatory factor X-associated protein (RFXAP) and major histocompatibility complex class II (MHC classII), which contribute to immune tolerance, has been observed to be suppressed in dendritic cells by miR-212-3p transported in exosomes released by pancreatic cancer cells [32]. 

The exchange of information between the microenvironment and tumor cells plays a key role in tumor progression. Exosomal miRNAs are able to make the microenvironment favorable or unfavorable to tumor growth by reprogramming the cells that constitute it. This explains their crucial role in cancer progression and metastasis. Bhome and colleagues showed that exosomal miR-21 produced by fibroblasts promotes CRC metastasis [33]. Prostate cancer-derived exosomal miR-940 is known to induce osteoblastic lesion in bone microenvironments, promoting metastasis [34].

## 5. Exosomal MiRNAs in Pediatric Cancers 

### 5.1. Neuroblastoma

Neuroblastoma (NB) is the most common extracranial solid tumor in children. This tumor derives from the neural crest and is characterized by a highly heterogeneous clinical behavior. *MYCN* amplification is one of the most important prognostic factors. Haug and coworkers analyzed exosomal miRNA profiles of nano-sized extracellular vesicles (EVs) isolated from *MYCN*-amplified NB cells (SK-N-BE(2)C and Kelly cells) [35]. Among the 25 highest expressed miRNAs in exosomes, 11 were common to both cell lines (miR-92a-3p, miR-23a-3p, miR-218-5p, miR-320a, miR-24-3p, miR-27b-3p, miR-16-5p, miR-25-3p, miR-21-5p- miR-125b-5p, and miR-320b) and several were oncogenic. A functional enrichment analysis using predicted mRNA target genes from the 25 highest expressed miRNAs revealed the Aryl hydrocarbon receptor (AHR) as the main signaling pathway. This signaling has been demonstrated to be involved in several aspects of cancer such as survival, apoptosis, differentiation, angiogenesis, and invasion [36]. This observation is in line with the suggestion that AHR is an upstream regulator of *MYCN*: ectopic overexpression of AHR suppressed *MYCN* promoter activity resulting in downregulation of *MYCN* expression, while AHR shRNA promoted the expression of *E2F1* and *MYCN* in NB cells [37]. In 2015, Challagundla and colleagues also published a work illustrating a possible role for exosomal miRNAs in the development of drug resistance through the involvement of the tumor microenvironment [38]. They showed that NB (SK-N-BE(2), CHLA-255, and IMR-32) cells transfer miR-21 to human monocytes via exosomes, inducing a pro-inflammatory effect through the activation of the NF-kB and TLR8 pathways in recipient cells. In turn, monocytes produce interleukin (IL)-6, which leads to STAT3 activation and consequent secretion of miR-155 within exosomes that are internalized by NB cells. In NB cells, miR-155 targets telomeric repeat binding factor-1 (TERF1), a component of the shelterin complex and inhibitor of telomerase, affecting telomere length, which is a prognostic factor in NB. Interestingly, it has also been observed that telomere length and telomerase activity correlate with drug resistance, tumor aggressiveness, and poorer outcomes in various malignancies [39,40,41]. In NB, the interplay between immune cells, the tumor microenvironment, and cancer cells contributes to immune escape and drug resistance. Natural killer (NK)-cell-derived exosomes carrying the tumor suppressor miR-186 induced cytotoxicity when internalized by *MYCN*-amplified NB cell lines, through an inhibitory action on *MYCN* expression, Aurora Kinase A (AURKA), and transforming growth factor (TGF)-β-receptors 1–2 [42]. The evaluation of miR-186 expression in high-risk NB patients showed a downregulation of this miRNA, confirming its role as a tumor suppressor. Recently, exosomal miRNA profiles have been performed on exosomes isolated from serum of 17 NB patients. This analysis showed 3779 exosomal miRNAs differentially expressed with respect to healthy controls (HCs). In particular 3248 were up- and 531 were downregulated. MiR-199a-3p, one of the most deregulated miRNAs in exosomes, was observed to correlate with disease severity [43]. In vitro upregulation of exosomal miR-199a-3p significantly increases proliferation and migration of NB cells, probably due to inhibition of the oncosuppressor neural precursor cell-expressed developmentally downregulated gene 4 (NEDD4).

### 5.2. Hepatoblastoma

Hepatoblastoma (HB) is a malignant embryonal liver tumor primarily affecting infants and very young children [44]. The pathogenesis of this malignancy is related to alterations in liver organogenesis, and often, the tumor recapitulates the stages of liver development [45]. Some genetic syndromes, such as Beckwith–Wiedemann syndrome, hemihypertrophy, and familial adenomatous polyposis (FAP), present an increased predisposition for HB [46]. Even with continuous improvements in the treatment of HB, the outcome for those patients with advanced disease who are refractory to preoperative chemotherapy remains unfavorable. The identification of effective biomarkers for early diagnosis, especially in patients who have genetic predisposition to developing HB, would be helpful a performing a prompt treatment and in helping to improve the outcome. Exosomal miRNAs are arousing great interest as possible biomarkers also in HB. Jiao and colleagues found that levels of exosomal miR-34a/b/c were significantly lower in serum of 89 patients with HB compared with HC groups [47]. Deregulation of miRNA-34 has been described to have a role in the promotion of several tumors such as lung, skin, breast, urinary bladder, and kidney [48,49] and may be considered as a diagnostic and prognostic biomarker in HB. Wanbo and coworkers showed that expression of miR-21 was higher in patients with HB compared with HCs in both plasma and exosomes. Exosomal miR-21 could be also considered a diagnostic and prognostic biomarker for patients with HB [50].

### 5.3. Osteosarcoma

Osteosarcoma (OS) is the most frequent malignant bone tumor, mainly affecting long bones of children and adolescents [51,52]. Its incidence has a bimodal age distribution: an initial peak at 10–14 years of age, which is consistent with the age of bone growth, and a second peak after 60 years [53]. At diagnosis, 10–15% of patients showed metastases, mainly in the lungs [54].

Although the long-term survival of nonmetastatic OS patients has been significantly improved over the last years [55], the risk of relapse or distant metastasis remains high. In this context, it would be useful to develop novel strategies for diagnosis, risk assessment, and personalized therapy in these patients.

To identify novel biomarkers of aggressiveness, several studies have investigated the expression profile of miRNAs carried in exosomes isolated from OS cells and/or plasma samples of patients.

Exosomal miR-675 was significantly upregulated in metastatic (MG63.2 and 143B) OS cell lines compared to nonmetastatic (MG63, HOS) cells [56]. Similarly, in plasma of lung metastatic patients, a significant upregulation of miR-675 was also observed compared to the nonmetastatic ones [56]. Interestingly, miR-675 is involved in migration, proliferation, and survival in multiple types of cancer [57,58]. Furthermore, this miRNA has been implicated in carcinogenesis and metastasis in gastric cancer by targeting Calneuron 1 (CALN1), a migration-related gene [59]. MiRNA target prediction analysis has confirmed that, also in OS, CALN1 is a target of miR-675. This observation was supported by in vitro experiments showing a downregulation of CALN1 expression in human osteoblast cell lines treated with exosomes enriched with miR-675 [56]. Indeed, lower levels of CALN1 and higher levels of exosomal miR-675 have been found in serum and in tumor tissue specimens of patients with OS and correlated with the presence of lung metastasis. This observation supports the hypothesis that this miRNA could be associated with metastasis [56]. Next generation sequencing (NGS) approach has been used to detect miRNAs enriched in EVs isolated from six different OS cell lines (SAOS2, MG63, HOS, 143B, U2OS, and hFOB1.19). Among 300 miRNAs identified, 4 miRNAs (miR143-3p, miR21-5p, miR181a-5p, and miR148-5p) were upregulated more than twofold in metastatic cell lines compared to nonmetastatic ones. Gene Ontology (GO) analysis suggested a role in transcriptional regulation of proteins involved in apoptosis, cell adhesion, and migration [60]. The major challenge in the treatment of OS patients is represented by chemotherapy resistance, which can favor the rapid growth of metastatic lesions [61]. The analysis of exosomal miRNAs in serum of OS patients with a poor response to chemotherapy identified deregulated exosomal miRNAs that are associated with OS progression. In particular miR-124, miR-133a, miR-199a-3p, and miR-385 were downregulated while miR-135b, miR-148a, miR-27a, and miR-9 were upregulated. Statistical analysis confirmed the diagnostic validity of these miRNAs as markers of treatment response, and DIANA_miRPath showed 30 KEGG biological processes associated with cancer that were significantly enriched in poor-response patients [62]. Tumor microenvironment has been documented to be strongly associated with tumor initiation and progression in OS and to contribute to poor prognosis of patients. Among different components, cancer-associated fibroblasts (CAFs) have been demonstrated to affect tumor cell properties such as proliferation, motility, drug resistance, and epithelial-to-mesenchymal transition [63,64]. Intracellular communication between tumor and CAFs can occur through exosomes. Exosomes derived from CAFs are able to promote migration and invasion of OS cells through the shuttling of miR-1228, which has oncogenic function in various cancers [65,66], and downregulation of its target, suppressor of cancer cell invasion (SCAI) [67]. Angiogenesis represents a crucial step for tumor growth, invasion, and metastatic dissemination. A high content of miR-25-3p was found in exosomes derived from the U2OS, OS, 143B, and SaOS2 OS cell lines. When added to HUVEC cells, exosomal miR-25-3p promoted capillary formation [68]. In OS tissues, dysregulation of miR-25-3p expression is correlated with poor prognosis [69], and previous works showed both oncogenic and tumor-suppressor functions depending on the cellular context [70,71]. To determine if cell lines and derived exosomes show the same expression pattern of miRNAs, Raimondi and colleagues performed small RNA sequencing on cells and related derived exosomes of the SAOS-2, MG-63, and U-2 OS cell lines. Data analysis showed that 21 human miRNAs were significantly differentially expressed between exosomes and the parental cell lines. Among those, 10 miRNAs were downregulated in exosomes (hsa-let-7b-5p, hsa-let-7d-3p, hsa-let-7e-5p, hsa-miR-23a-5p, hsa-miR-214-3p, hsa-miR-125a-5p, hsa-miR-331-3p, hsa-miR-193b-3p, hsa-miR-941, and hsa-miR-1908-5p) and 11 were upregulated (hsa-let-7f-5p, hsa-miR-16-5p, hsa-miR-21-5p, hsa-miR-192-5p, hsa-miR-148a-3p, hsa-miR-182-5p, hsa-miR-128-3p, hsa-miR-126-5p, hsa-miR-186-5p, hsa-miR-301a-3p, and hsa-miR-151a-3p). They observed that miR-21-5p and miR-148a, known to be involved in bone remodeling and neo-angiogenesis, were significantly increased in Raw264.7 and HUVEC cells, respectively, when treated with OS cell-derived exosomes. Moreover, while the overexpression of these 2 miRNAs in Raw264.7 cells induced expression of osteoclast markers and stimulated bone resorption activity, in HUVEC cells, it promoted angiogenesis [72]. Altogether, these findings suggest a potential role for these miRNAs as prognostic biomarkers in OS.

### 5.4. Ewing’s Sarcoma

Ewing’s sarcoma (EWS) is the second most common primary bone cancer [73]. EWS is a predominant childhood malignancy, histologically characterized by small round cells with high levels of the membrane glycoprotein cluster of differentiation (CD) 99. This tumor is characterized by balanced chromosomal translocations t(11;22)(q24;q12), which results in the production of EWS-FLI1 oncoprotein. EWS cells can release CD99 through exosomes. Analyzing the specific cargo loaded in these exosomes, Ventura et al. focused on miRNA content and identified factors that affect malignancy and that shape the genetic landscape of EWS neoplastic cells. Both CD99 and EWS-FLI1 appear to impact EWS cell differentiation with opposite effects: while EWS-FLI1 may induce neural differentiation, CD99 prevents it [74]. The inhibition of differentiation by CD99 takes place through nuclear factor kappa-light-chain-enhancer of activated B cells (NF-kB) signaling, which is regulated by the miR-34a-induced Notch pathway. Interestingly, comparison of CD99-silenced with wild-type EWS cells showed differences in exosome secretion. Indeed, deficiency of CD99 increased miR-34a and decreased Notch 1 and Notch 3 levels. Moreover, exosomes isolated from CD99-silenced EWS cells were able to influence the recipient wild-type EWS cells by mimicking CD99 silencing and by inducing neural differentiation. This effect is probably related to downregulation of NF-kB signaling due to an internalization of exosomes carrying miR-34a. Thus, miR-34a contained in exosomes could actively participate in the tumorigenesis of EWS [74]. A different group identified miR-199-3p as the most enriched miRNA inside CD99-silenced exosomes [75]. This miRNA has been implicated in the suppression of tumor growth, migration, and invasion and has the ability to reduce EWS malignancy in in vitro experimental models. Moreover, the expression of miR-199a-3p was significantly lower in metastases than in localized primary tumors, suggesting its involvement in EWS aggressiveness [75].

### 5.5. Rhabdomyosarcoma

Rhabdomyosarcoma (RMS) is the most common soft tissue sarcoma in children and young adults [76], accounting for 5–10% of all pediatric malignancies. On the basis of histology, RMS shows two subtypes: the alveolar (ARMS) and the embryonal RMS (ERMS) [77]. ERMS is composed of cells similar to immature skeletal myoblasts and represents approximately 75% of RMS [78]. ARMS is present in around 25% of patients, appears histologically similar to pulmonary parenchyma, and frequently presents metastatic dissemination at diagnosis with poor outcome [79]. The ARMS variant is characterized by a chromosomal translocation t(2;13) (q35;q14), resulting in the fusion of the gene paired box (*PAX*)-3 with the gene forkhead box protein O1 (*FOXO1*) on chromosome 13. In a minor proportion of ARMS, the chromosomal translocation t(1;13) (p36;q14) is present, which results in a fusion between PAX7 on chromosome 1 and the FOXO1 gene [80].

Recently, a role in proliferation, migration, and angiogenesis of exosomal miRNAs to support growth has been reported also in RMS. Gayad and colleagues evaluated exosomal miRNAs derived from 3 ERMS (JR1, RD, and RH36) and 2 ARMS (RH30 and RH41) and showed that miRNAs found in ARMS exosomes clustered together and were exclusive when compared to ERMS exosomes. In particular, 34 miRNAs were enriched in JR1 and RD, while 62 miRNAs were present exclusively in ARMS-derived exosomes. Ten miRNAs were common among the two subtypes, excluding RH36, while only 2 miRNAs (miR-1246 and miR-1268) were present in exosomes of all cell lines. Target scan software and PANTHER analysis revealed that these two miRNAs are involved in several pathways related to tumorigenesis (Wnt, Cadherin, epidermal growth factor, and fibroblast growth factor), angiogenesis, and apoptosis [22]. Functional analysis revealed that RMS exosomes increased proliferation of human fibroblasts and of the same cancer cells: exosomes derived from ARMS cells influence proliferation of ERMS cells and vice versa. Moreover, as tumor-associated fibroblasts have been shown to play a pivotal role in local invasion and metastasis in solid tumors [81], the authors investigated the effect of RMS-derived exosomes on invasive and migratory properties on normal fibroblasts and observed a significant increase in migration. Furthermore, RMS-derived exosomes stimulated angiogenesis as an increased ability of HUVEC cells to differentiate into capillary-like structures when plated on matrigel [22].

### 5.6. NRSTS (Non-Rhabdomyosarcoma Soft Tissue Sarcoma)

NRSTS accounts for about 3–4% of pediatric cancers and constitutes a very heterogeneous group of tumors with different behaviors, genetics, and clinical presentations [82]. Synovial sarcoma (SS) is the most common malignant NRSTS in children and adolescents, representing approximately 5–10% of all soft tissue sarcomas. SS is characterized by local invasiveness and has a propensity to metastasize to the lung, lymph nodes, and bone marrow [83]. Most cases of SS display the t (X; 18) (p11.2; q11.2) chromosomal translocation, resulting in the fusion of the SYT gene on chromosome 18 with the SSX1 or the SSX2 gene located on chromosome X [84]. The prognosis for SS patients depends largely on tumor size and site, presence of any metastases, and feasibility of surgical resection. Due to the rarity of this tumor, the optimal treatment of SS remains to be established. SS has traditionally been considered sensitive to chemotherapy based on anthracyclines and ifosfamide; however, therapeutic options are limited. The multitarget tyrosine kinase inhibitor (TKI) Pazopanib has showed both antiangiogenesis and antitumor effects on SS cells [85]. However, the efficacy of this TKI is often limited by pazopanib resistance [86]. Shiozawa et al. showed that EVs released by pazopanib treated/untreated SS cell lines (SYO-1, HS-SYII, 1273/99, and YaFuSS) have increased levels of miR-761 [87]. This miRNA positively correlates with treatment resistance by targeting thyroid hormone receptor interactor 6 (TRIP6), lamin A/C (LMNA), and sirtuin 3 (SIRT3). MiRNA profiling analysis using serum from SS patients and medium from SS cell lines showed a significant upregulation of miR-92b-3p. Interestingly, expression level analysis showed that this miRNA is present at a higher level in SS-cell (SYO-1, HS-SY-II, and YaFuSS)-derived exosomes than in human mesenchymal stromal cell (MSC)-derived exosomes. MiR-92b-3p has been reported to be specifically overexpressed in primary brain tumors [88] and to regulate the development of intermediate cortical progenitors in embryonic mouse brain [89]. The presence of this miRNA in exosomes released from SS cells suggests a possible neuroectodermal origin of this neoplasm [90].

Desmoplastic small round cell tumor (DSRCT) is an aggressive mesenchymal tumor affecting primarily adolescent and young adult males. DSRCT typically presents with large abdominal mass already widely disseminated at the time of diagnosis and has an extremely poor outcome despite the use of intensive multimodality treatment approaches. This malignancy is associated with a typical chromosomal translocation, t(11;22) (p13;q12), involving a fusion between the *EWSR1* and *WT1* gene, which leads to production of chimeric protein with transcriptional regulatory activity [91]. Due to the rarity of this malignancy, biomarkers for diagnosis, treatment stratification, and prognosis are poorly studied and defined. Recently, our group investigated the exosomal miRNA profiles in plasma samples derived from three patients with DSRCT [92] compared with HCs. Fifty-five miRNAs were identified to be significantly modulated in DSRCT-derived exosomes with respect to HC-derived exosomes. Among these 55 miRNAs, 14 were highly dysregulated in at least one patient and only 5 were expressed in all three patients: miR-34a-5p, miR-22-3p, and miR-324-3p were upregulated while miR-342-3p and miR-150-5p were downregulated in exosomes isolated from DSRCT patients with respect to that obtained from HCs. Interestingly, these miRNAs are modulated in several cancers where they have a key role in cell growth, proliferation, migration, and invasion of cancer cells. Our analysis emphasizes that the miRNAs upregulated in DSRCT-derived exosomes have a tumor suppressive function, and in this way, the tumor cell promotes its oncogenic potential. On the other hand, exosomal miRNAs upregulated with respect to HCs and released from the tumor may act to enhance the oncogenic potential of cancer cells. Moreover GO categories and pathway analyses showed that the MAPK and Rap1 signaling pathways, which are involved in tumor progression, were targeted by the three miRNAs upregulated in all three patients with DSRCT and that a large number of transcripts targeted by the highly dysregulated miRNAs are involved in the intracellular receptor signaling pathway and in nervous system development.

### 5.7. Brain Tumors

Primary tumors of the central nervous system (CNS) account for the second most frequent malignancy in children. Among these tumors, gliomas are the most common entity, and pediatric high-grade gliomas (pHGG), including diffuse intrinsic pontine glioma (DIPG), represent rapidly lethal malignancies [93].

In the last years, molecular profiling revolutionized our understanding of pHGG biology. However, the intratumor heterogeneity is a complicating factor for the treatment of pHGG, and novel approaches are required to overcome this limitation. For these reasons, the identification of biomarkers that reflect the tumor heterogeneity and that allow the monitoring of disease progression or response to treatment is needed. Exosomes are emerging as a source of possible biomarkers in the context of brain tumors. Tűzesi and collaborators isolated exosomes released by pediatric glioma stem cells (GSCs) and compared their miRNA content with exosomes released from normal neuronal stem cells (NSCs) [94]. They found that 37 and 5 miRNAs were respectively up- and downregulated in the GSC with respect to NSC exosomes. Among the upregulated miRNAs, miR-1290 and miR-1246 have roles in stemness and cancer progression [94]. Enriched KEGG pathways targeted by miRNAs differentially expressed, such as choline metabolism, proteoglycans, and glycosphingolipids biosynthesis, suggest a putative role in cancer-related pathways. Furthermore, they identify differentially expressed miRNAs between cells and exosomes (152 miRNAs between NSCs and their exosomes and 196 for GSCs). Most of these miRNAs were upregulated, suggesting a specific role for miRNA packaging into exosomes produced by glioma cells. Moreover, exosomes released by GSC cells can promote tumorigenesis in NSC recipient cells altering genes expression. In particular downregulation of glioma-associated tumor suppressor phosphatase and tensin homolog (PTEN) and Tet methylcytosine dioxygenase 3 (TET3) and upregulation of cancer-related genes such as SERTA domain-containing protein 1 (SERTAD1) and protein transport protein Sec61 subunit gamma (SEC61G) have been observed in NSC cells after treatment with exosomes isolated from GSC cells [94].

Atypical teratoid/rhabdoid tumor (ATRT) is a rare, high-grade embryonal pediatric brain tumor with high aggressiveness and very poor overall survival. Despite the advances in cancer biology and treatment, much remains unknown about the molecular mechanisms involved in ATRT growth and development and the standard therapy is usually ineffective. Microenvironment cells, such as MSCs, tumor-associated MSCs (tMSCs), and endothelial cells, appear to have a role in the regulation of the stem component of this tumor. In particular, it has been observed that miR-155 contained in exosomes isolated from tMSCs is able to increase the migratory ability of ATRT cells by directly targeting *SMARCA4* [95]. On the other hand, ATRT cells stimulate tMSCs to release a higher number of exosomes, further improving migration of ATRT cells.

### 5.8. Leukemias

Leukemias are the most common cancer in children, accounting for about 30% of all tumors. Among them, acute lymphocytic leukemia (ALL) is more frequent than acute myeloid leukemia (AML), while only rare cases present chronic myeloid leukemia and juvenile myelomonocytic leukemia [96,97,98,99,100,101,102]. AML is characterized by high proliferation and subsequent accumulation of immature myeloid precursor cells in the bone marrow or in the blood, while ALL arises from clonal proliferation of early B and T lymphocyte progenitors, resulting in accumulation of blasts in bone marrow and also in extramedullary sites [103,104,105]. In recent years, several studies have supported the role of miRNAs in the pathogenesis of pediatric leukemias, suggesting their potential activity in these cancers [106]. Higher expression of miR-128a, miR-34, miR-142, and miR-18b and downregulation of miR-100, miR-196b, miR-125a-5p, and let-7e have been found in ALL [107,108,109,110,111], whereas lower expression of miR-29b-1 and miR-223 and upregulation of miR-196b and miR-155 were related to AML prognosis [112,113,114,115,116,117]. Most recent works describe a high number of modulated miRNAs in AML also based on high-risk of disease, treatment resistance, and patient survival, suggesting miRNAs as potential biomarkers in pediatric leukemias [118,119,120]. Circulating miR-150, miR-342, miR-370, and miR-335 were found down- and upregulated in AML; downregulation of miR-146a and upregulation of miR-100 and miR-196a were described in ALL [103,105,121]. Although a growing list of circulating miRNAs has been discovered, there is less information about exosomal miRNAs. Leukemic cells produce exosomes which are involved in the reprogramming of the bone marrow (BM) microenvironment, drug resistance, and relapse by inhibiting the antileukemia immunity [122,123,124,125,126,127,128,129,130,131,132,133]. In particular, stroma, angiogenesis, and mesenchymal stromal cell proliferation are influenced by the transfer of exosomal miRNAs through the reprogramming of the BM microenvironment [128,129]. Among these, miR-155, miR-375, miR-150, miR-92a, miR-210, miR-29, miR-223, miR-202-3p, miR-21, miR-146a, miR-148, and mi-135b were specifically reported [129,130,131,132,133,134,135]. Hornick et al. described a panel of overexpressed serum exosomal miRNAs from NOD scid gamma (NGS) mice serum as possible early biomarkers of AML: let-7a, miR-99b, miR-146a, miR-150, miR-155, miR-191, and miR-1246 [136]. The involvement of exosomal miRNAs in pediatric leukemias has been poorly investigated, and more studies are needed to characterize them and to assess their effects.

### 5.9. Lymphomas

Lymphoma affects the lymphatic system and accounts for about 15% of pediatric malignancies [98,137]. It is caused by malignant transformation of lymphocytes with lymph node involvement, but it can also affect the bone marrow and other organs. Lymphomas are classified according to the World Health Organization (WHO) system [138], which identifies these neoplasms derived from precursor lymphoid or mature lymphoid cells and distinguishes each group on the basis of B-cell or T-cell origin [137,139]. Hodgkin lymphoma (HL also called Hodgkin disease) and non-Hodgkin lymphoma (NHL) represent the two main categories of lymphoma. Both types occur in children and adults. Hodgkin’s disease develops from a specific abnormal B lymphocyte lineage, while NHL may derive from either abnormal B or T cells and are distinguished by unique genetic markers. HL is more common in early and late adulthood, but it is rare in children younger than 5 years of age. NHL makes up about 5% of childhood cancers and is more likely to occur in younger children than HL, but it is still rare in children younger than 3 years old [140]. Burkitt lymphoma (BL), diffuse large B-cell lymphoma (DLBCL), primary mediastinal large B-cell lymphoma (PMLBCL), anaplastic large cell lymphoma (ALCL), and lymphoblastic lymphoma (LL) are included in NHLs [141]. Aberrant miRNA expression has been found in different lymphomas; some of these are known for their pathological role. Most of the studies about exosomal miRNAs in lymphomas are focused on adults; thus, so far, there is no information in pediatric settings. Considering the high amount of promising data in adults, it would be interesting to test whether they apply to these diseases in childhood. Mir-155 is encoded by the human BIC gene that is overexpressed in pediatric BL [142]; however, miR-155 was found expressed only in BL EBV positive cases, which account for 70% of all pediatric BL [143,144]. It was demonstrated that exosomes from EBV-positive Raji cells deliver miR-155 to retinal epithelial pigment cells (ARPE-19), modulating transcription and translation levels of VEGF-A [145]. Upregulation of miR-17-3p, miR-92, miR-516-3p, miR-520d, miR-573, miR-595, miR-629, and miR-663 was also identified in B-cell lymphomas [146,147,148]. Zhuang et al. suggested a role of miR-146a in the pathogenesis of DLBCL because of its higher level of expression in blood samples of patients than healthy donors [149]. However, Zare et al. showed that, in DLBCL patients, no significant difference in the expression level of exosomal miR-146a correlated to therapy response, but only an accumulation of this miR in the exosomes was compared to the free plasma levels [150]. Kare et al. also performed a profile of miRNAs expressed in whole plasma and in exosomes derived from adult patients affected by DLBCL [151]. They showed the overexpression of miR-124 and miR-532 and the underexpression of 19 miRNAs (miR-122, miR-128, miR-141, miR-145, miR-197, miR-345, miR-424, miR-425, miR-101, miR-324, let-7e, miR-222, miR-29c, miR-375, miR-324-5p, miR-135a, miR-379, let-7i*, and miR-32) in 14 DLBCL patients compared to 20 healthy donors. Exosomal miRNAs were also found deregulated in DLBCL in response to chemotherapy. In particular, exosomal miR-99a-5p and miR-125b-5p were significantly higher in the serum of chemoresistant patients with DLBCL compared to the chemosensitive group and were associated with shorter progression-free survival [152].

## 6. Conclusions

**Figure 1:**
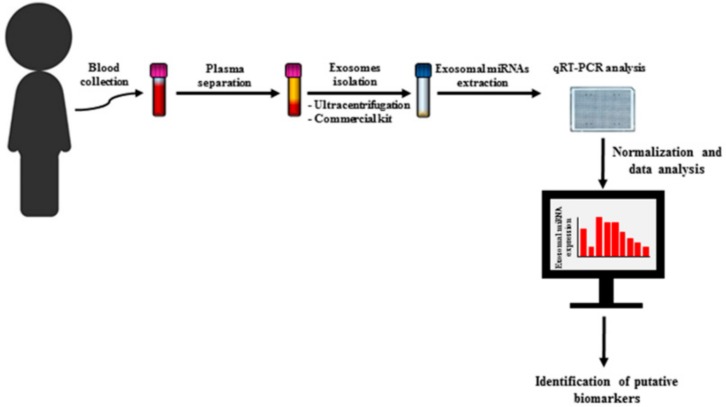
Identification of exosomal miRNAs as biomarkers in pediatric cancer: Exosomal miRNAs display different expression patterns between cancer patients and healthy children. Moreover, their expression changes during disease progression and after therapy and can therefore be promising biomarkers. With a simple venipuncture, the blood sample is collected and processed to separate the plasma or serum through serial centrifuges. Exosomes from serum or plasma are then isolated by ultracentrifugation or by using commercial kits. The exosomes pellet is lysate, and RNA is extracted. After retro-transcription, miRNA expression is assessed by real-time PCR and the data obtained is analyzed with specific software for identification of statistically deregulated miRNAs which may have a possible role as biomarkers.

The current challenges in the field of childhood cancer diseases include the identification of novel biomarkers that may allow noninvasive diagnosis, risk stratification, and follow-up. Even though numerous advances have been made at the diagnostic and therapeutic levels in pediatric oncology, the diagnosis is still based on the symptomatology that often becomes evident only in the late stages. The emerging involvement of miRNA deregulation in the cancer pathogenesis has opened promising opportunities for their clinical application in tumor diagnosis, outcome prediction, and therapy. Moreover, the discovery that miRNAs can be loaded and transported into nanovesicles called exosomes released from cells in body fluids have shown the possibility of using exosomal miRNAs as noninvasive biomarkers (Table 1). It is now known that exosomes are released in greater numbers by tumor cells and that their content differs in several pathologies compared to a physiological condition. Furthermore, exosomal miRNA content changes during cancer progression and in response to therapy. Advances in isolation techniques of these EVs from biological fluids and analysis of their content, particularly miRNAs, makes them particularly interesting for monitoring the disease relatively quickly and without intervening with invasive techniques (Figure 1). Although many aspects still need to be clarified on the specific role of exosomes in the pediatric setting, exosomal miRNA could represent clinically feasible disease biomarkers in pediatric cancers.

## Figures and Tables

**Table 1:** MicroRNAs (MiRNAs) contained in pediatric tumor exosomes.

Pediatric Tumor	Site of Exosomes	miRNA	Upregulated/ Downregulated	Function	References
**Neuroblastoma**					
SK-N-BE(2)C and Kelly cells	Culture Media	miR-92a-3p, miR-23a-3p, miR-218-5p, miR-320a, miR-24-3p, miR-27b-3p, miR-16-5p, miR-25-3p, miR-21-5p- miR-125b-5p, an miR-320b	Upregulated	Aryl hydrocarbon receptor (AHR) signaling pathway, survival, apoptosis, differentiation, angiogenesis, and invasion	[35]
SK-N-BE(2)C, CHLA-255, and IMR-32	Culture media	miR-21	Upregulated	Pro-inflammatory effect through the activation of the NF-kb and TLR8 pathways	[38]
	Plasma	miR-199a-3p	Upregulated	Increased proliferation and migration	[43]
**Hepatoblastoma**					
	Serum	miR-34a, miR-34b, and miR-34c	Downregulated	Tumor promotion	[47]
	Plasma	miR-21	Upregulated		[50]
**Osteosarcoma**					
MG63, MG63.2, HOS, and 143B cells	Culture Media Plasma	miR-675	Upregulated	Migration, proliferation, and survival	[56]
SAOS2; MG63; HOS; 143B; U2OS, and hFOB1.19 cells	Culture media	miR143-3p, miR21-5p, miR181a-5p, and miR148-5p	Upregulated	Apoptosis, cell adhesion, and migration	[60]
	Serum	miR-135b, miR-148a, miR-27a, and miR-9	Upregulated		[62]
		miR-124, miR-133a, miR 199a-3p, and miR-385	Downregulated		
U2OS, HOS, 143B, and SAOS2 cells	Culture media	miR-25-3p	Upregulated	Promotes capillary formation	[68]
SAOS-2, MG-63, and U-2 OS cells	Culture media	hsa-let-7f-5p, hsa-miR-16-5p, hsa-miR-21-5p, hsa-miR-192-5p, hsa-miR-148a-3p, hsa-miR-182-5p, hsa-miR-128-3p, hsa-miR-126-5p, hsa-miR-186-5p, hsa-miR-301a-3p, and hsa-miR-151a-3p	Upregulated		[72]
		hsa-let-7b-5p, hsa-let-7d-3p, hsa-let-7e-5p, hsa-miR-23a-5p, hsa-miR-214-3p, hsa-miR-125a-5p, hsa-miR-331-3p, hsa-miR-193b-3p, hsa-miR-941, and hsa-miR-1908-5p	Downregulated		
**Ewing Sarcoma (EWS)**					
EWS cells	Culture media	miR-34a	Upregulated	Downregulates NF-k B signaling	[74]
CD99neg-EWS cells	Culture media	miR-199-3p	Upregulated	Suppression of tumor growth, migration, and invasion	[75]
**Rhabdomyosarcoma**					
RH30, RH41, RD, JR1, and RH36 cells	Culture Media	miR-1246 miR-1268		Tumorigenesis, angiogenesis, and apoptosis	[22]
**Synovial Sarcoma**					
SYO-1, HS-SYII, 1273/99, and YaFuSS cells	Culture Media	miR-761	Upregulated	Positively correlated with treatment resistance	[87]
SYO-1, HS-SY-II, and YaFuSS cells	Culture Media Serum	miR-92b-3p	Upregulated		[90]
**Desmoplastic small round cell tumor (DSRCT)**					
	Plasma	miR-34-5p miR-22-3p miR-324-3p	Upregulated	Cell growth, proliferation, migration, and invasion	[92]
		miR-342-3p miR-150-5p	Downregulated		
**Brain tumors**					
GSCS cells	Culture Media	miR-1290 miR-1246	Upregulated	Stemness and cancer progression	[94]

## Abbreviations

mRNA	Messenger RNA
RISC	RNA-induced silencing complex
ESCRT	Endosomal sorting complex required for transport
TSG101	Tumor-susceptibility gene 101
ALIX	ALG-2 interacting protein X
MVB	Microvesicular bodies
SMase	Sphingomyelinase
hnRNP	Heterogeneous nuclear ribonucleoproteins
miRISC	miRNA-induced silencing complex
AGO2	Argonaute protein 2
CRC	Colon rectal cancer
HUVEC	Human umbilical vascular endothelial cells
TMZ	Temozolomide
GBM	Glioblastoma
XRCC4	X-ray repair cross-completing 4
ER	Estrogen receptor
M	Macrophage
MAPK	Mitogen-activated protein kinase
STAT	Signal transducer and activator of transcription
RFXAP	Regulatory factor X-associated protein
MHC class II	Major histocompatibility complex class II
NB	Neuroblastoma
AHR	Aryl hydrocarbon receptor
IL	Interleukin
TERF1	Telomeric repeat binding factor-1
NK	Natural killer
AURKA	Aurora Kinase A
TGF	Transforming growth factor
HC	Healthy control
NEDD4	Neural precursor cell-expressed developmentally downregulated gene 4
HB	Hepatoblastoma
FAP	Familial adenomatous polyposis
OS	Osteosarcoma
CALN1	Calneuron-1
NGS	Next generation sequencing
EV	Extracellular vesicle
GO	Gene ontology
CAF	Cancer-associated fibroblast
SCAI	Suppressor of cancer cell invasion
EWS	Ewing’s sarcoma
CD	Cluster of differentiation
NF-kB	Nuclear factor kappa-light-chain-enhancer of activated B cells
RMS	Rhabdomyosarcoma
ARMS	Alveolar rhabdomyosarcoma
ERMS	Embryonal rhabdomyosarcoma
PAX	Paired box
FOXO1	Forkhead box protein O1
NRSTS	Non-rhabdomyosarcoma soft tissue sarcoma
SS	Synovial sarcoma
TKI	Tyrosine kinase inhibitor
TRIP6	Thyroid hormone receptor interactor 6
LMNA	Lamin A
SIRT3	Sirtuin 3
MSC	Mesenchymal stromal cell
DSRCT	Desmoplastic small round cell tumor
CNS	Central nervous system
pHGG	Pediatric high-grade glioma
DIPG	Diffuse intrinsic pontine glioma
GSCs	Pediatric glioma stem cells
NSCs	Neuronal stem cells
PTEN	Phosphatase and tensin homolog
TET3	Tet methylcytosine dioxygenase 3
SERTAD1	Serta domain-containing protein 1
ATRT	Atypical teratoid/rhabdoid tumor
ALL	Acute lymphocytic leukemia
AML	Acute myeloid leukemia
BM	Bone marrow
NGS	Nod scid gamma
WHO	World Health Organization
HL	Hodgkin lymphoma
NHL	Non-Hodgkin lymphoma
BL	Burkitt lymphoma
DLBCL	Diffuse large B-cell lymphoma
PMLBCL	Primary mediastinal large B-cell lymphoma
ALCL	Anaplastic large cell lymphoma
LL	Lymphoblastic lymphoma
